# Bovine pericardial patch for preventing air leak after thoracoscopic-assisted pulmonary wedge resection: a retrospective cohort study with predictive modeling

**DOI:** 10.3389/fonc.2026.1790677

**Published:** 2026-03-30

**Authors:** Fei-Long Guo, Wei-Ming Chen, Jun-Peng Zhan, Zhen-Yang Zhang, Jiang-Bo Lin, Sui Chen

**Affiliations:** 1 Department of Thoracic Surgery, Fujian Medical University Union Hospital, Fuzhou, China; 2 Key Laboratory of Cardio-Thoracic Surgery (Fujian Medical University), Fujian Province University, Fuzhou, China; 3 National Key Clinical Specialty of Thoracic Surgery, Fuzhou, China; 4 Clinical Research Center for Thoracic Tumors of Fujian Province, Fuzhou, China

**Keywords:** bovine pericardial patch, postoperative air leak, thoracoscopic wedge resection, predictive model, risk factors

## Abstract

**Background:**

Thoracoscopic wedge resection has become a standard procedure for pulmonary nodule management, yet postoperative air leak (PAL) remains a prevalent complication. While bovine pericardial patches are established in cardiovascular surgery, their utility in pulmonary resection merits further investigation. This study aimed to assess the efficacy of bovine pericardial patches in reducing PAL after thoracoscopic wedge resection and develop a predictive model for clinical utility.

**Methods:**

In this single-center retrospective study (2015-2020), we analyzed 2006 thoracoscopic wedge resections at Fujian Medical University Union Hospital, comparing 319 patch-treated cases with 1,687 controls. Primary outcomes included PAL incidence; while secondary outcomes encompassed drainage duration. Univariate and multivariate logistic regression analyses identified risk factors for PAL, and a nomogram was constructed to predict PAL risk.

**Results:**

Baseline characteristics were well-balanced between groups. The bovine pericardial patch group showed a significantly lower PAL incidence (2.8% vs. 13.2%, *P* < 0.001) and shorter chest tube drainage duration (2.85 ± 1.27 days vs. 3.06 ± 1.67 days, *P* = 0.033). Multivariate analysis confirmed the bovine pericardium patch as an independent protective factor against PAL (OR: 0.170; 95% CI: 0.079-0.322; *P* < 0.001). The nomogram incorporating these factors showed good discriminative ability (AUC = 0.739) and clinical utility in decision curve analysis.

**Conclusion:**

Bovine pericardial patch application significantly reduces PAL incidence and shortens chest tube drainage duration after thoracoscopic wedge resection. The predictive nomogram helps identify high-risk patients who would benefit most from this intervention, supporting its use as a cost-effective adjunct in pulmonary surgery.

## Introduction

Lung cancer maintains its status as the most prevalent malignancy globally, with persistently high incidence and mortality rates (1). The widespread adoption of low-dose computed tomography screening and enhanced public health awareness has substantially increased the detection of pulmonary nodules (2). Surgical resection remains the cornerstone of treatment for early-stage lung cancer and precancerous lesions (3, 4), with the volume of pulmonary surgeries demonstrating a steady annual increase (5). In this context, enhanced recovery after surgery and complication mitigation have emerged as critical research priorities.

Thoracoscopic wedge resection has become the procedure of choice for small pulmonary lesions, offering favorable outcomes when adequate margins can be achieved (6, 7). While this limited resection approach demonstrates lower complication rates compared to anatomical resections (8, 9), postoperative air leak (PAL) remains a significant concern, with reported incidence rates varying from 3.0% to 15% (10–12). Prolonged air leakage frequently necessitates additional interventions, including re-intubation or surgical re-exploration, substantially impacting patient recovery and healthcare costs (13).

Current preventive strategies exhibit inconsistent efficacy and cost-effectiveness. Lopez et al. (14) demonstrated that fibrin sealant application reduced both PAL duration and hospital stay, while Suzuki et al. (15) reported superior outcomes with combined fibrin glue and polyglycolic acid (PGA) patches. Previously reported materials for preventing postoperative air leak include PGA sheets, fibrin sealants, and TachoSil, which have demonstrated varying efficacy in reducing PAL rates following lung resection, though optimal strategies remain under investigation (15, 16). However, the high material costs associated with these approaches-particularly when using PGA patches-present substantial economic burdens (16). This underscores the need for cost-effective alternatives that maintain clinical effectiveness.

Bovine pericardial patches, widely employed in vascular reconstruction (17, 18), possess ideal biological properties including excellent sealing capacity and absorbability. Their potential application in pulmonary surgery remains underexplored, particularly regarding wedge resection outcomes. To our knowledge, no previous study has comprehensively evaluated (1): the efficacy of bovine pericardial patches in preventing PAL (2), their impact on multiple perioperative outcomes, and (3) predictive factors for successful implementation. Therefore, this retrospective study was performed to compare PAL incidence between patch-treated and control groups and to develop a predictive model identifying patients who would derive maximal benefit from patch application.

## Methods

### Study design and protocol

This single-center retrospective cohort study was conducted at Fujian Medical University Union Hospital after obtaining ethical approval from the Institutional Review Board (Approval No: 2024KY186). The requirement for informed consent was waived due to the retrospective nature of the study, which complied with the Declaration of Helsinki principles.

### Patients selection and data source

We analyzed clinical records of 2,006 consecutive patients (1,016 males; 990 females) who underwent thoracoscopic-assisted wedge resection between January 2015 and December 2020. Data were extracted from our prospectively maintained thoracic surgery database with additional chart review verification. Patients were included if they met: (1) Peripheral pulmonary nodules (≤3 lesions per side) confirmed by contrast-enhanced CT, with Nodule center in outer third of lung field, maximum diameter <2 cm, and No radiological evidence of lymphadenopathy/metastasis; (2) Adequate cardiopulmonary function for general anesthesia; (3) Complete wedge resection via thoracoscopic-assisted approach; (4) Negative intraoperative air leak test; and (5) Complete perioperative documentation. The exclusion criteria were wedge resections performed for bullous disease.

### Preoperative evaluation and surgical protocol

All patients underwent standardized preoperative assessment within 4 weeks, including laboratory tests (complete blood count, coagulation profile), pulmonary function tests, contrast-enhanced chest CT (nodule localization/staging), abdominal ultrasound and brain MRI (metastasis screening), multidisciplinary team evaluation for surgical indication, and 8th edition TNM staging for suspected malignancies.

The bovine pericardial patch was not applied routinely in all patients but was used selectively (319 cases) as a prophylactic reinforcement of the staple line during thoracoscopic wedge resection. Decisions were surgeon-dependent and could be influenced by individual surgeon experience, intraoperative findings (such as assessment of parenchymal fragility or staple-line tension), and patient characteristics not fully captured in our retrospective records. All procedures included an intraoperative air-leak test, and only cases with a negative test result were eligible for inclusion in the study.

**Figure 1:**
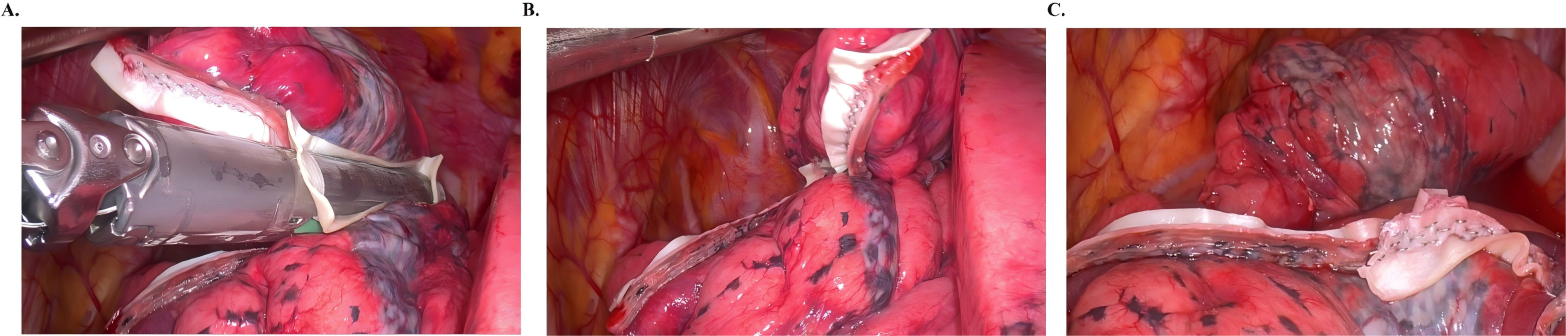
Schematic illustration of bovine pericardial patch application in thoracoscopic wedge resection. **(a)** bovine pericardial patch combined with a linear stapler for thoracoscopic wedge resection; **(b, c)** the resected lung stump is wrapped with the bovine pericardial patch.

Procedures were performed under general anesthesia with double-lumen intubation and single-lung ventilation. Key technical aspects: (1) Positioning: Lateral decubitus (non-operative side down); (2) Access: 3-cm utility incision at 4th/5th intercostal space (mid-axillary line) with wound protector; (3) Visualization: 10mm 30° thoracoscope through single port; (4) Nodule identification: Combined visual inspection (pleural changes) and digital palpation; (5) Resection technique: Electrocautery marking of resection margins, Wedge resection using endoscopic staplers (4.5mm staples, Ethicon) ± bovine pericardial patch (BalMedic), and Specimen retrieval in endoscopic bag (**Figure 1**); and (6) Margin assessment: ≥2cm margins for suspected malignancies and intraoperative frozen section analysis guiding further resection.

### Data collection and outcome definition

Comprehensive variables were collected including demographic/clinical characteristics (age, sex, body mass index [BMI], ASA score; comorbidities: hypertension, diabetes mellitus [DM], cardiovascular/pulmonary diseases; smoking/alcohol history; and prior thoracic surgeries), radiological parameter (Emphysema index, and nodule characteristics), and intraoperative details (patch utilization, operative time, blood loss, number of wedge resections, and pleural adhesions).

The primary endpoint was postoperative air leak (PAL). PAL was defined as continuous bubbling in the chest drainage system during intentional coughing that prevented chest tube removal within the first 24 postoperative hours. We selected this 24-hour threshold because it aligns with our Enhanced Recovery After Surgery (ERAS) protocol, which aims for early chest tube removal. Air leaks were assessed twice daily (during morning and evening rounds) by the attending surgeons. Chest tube removal was performed when no air leak was observed for 24 hours, drainage volume was <200 mL/day, and there was no evidence of chylothorax or active bleeding. Of note, all patients were managed with traditional water-seal drainage systems throughout the study period (2015–2020), as digital drainage devices were not routinely available at our center. The second outcome was duration of chest tube drainage.

### Statistical analysis

Categorical variables were presented as numbers (percentages) and compared using Pearson’s chi-square test. Fisher’s exact test was employed when expected frequencies were <5. Continuous variables were described as mean ± standard deviation if normally distributed or median (interquartile range) if non-normally distributed, with between-group comparisons performed using the Mann-Whitney U test. Univariate logistic regression was first conducted to identify potential risk factors associated with PAL. Variables with *P* < 0.10 in the univariate analysis were subsequently included in a multivariate logistic regression model to determine independent risk factors. Variable selection for the predictive model involved univariate logistic regression to screen candidate predictors with p < 0.05. Multivariable logistic regression with stepwise forward selection was then used to identify independent predictors of prolonged air leak (PAL). The final model included three variables (age ≥65 years, pleural adhesions as risk factors; bovine pericardial patch use as a protective factor), with approximately 220 PAL events in the cohort, resulting in about 73 events per variable. A nomogram was constructed based on the multivariable model. Internal validation was performed using the bootstrap method (1,000 resamples) to evaluate calibration (via calibration plots) and discrimination. All statistical analyses were performed using SPSS 20.0 (IBM Corp., USA) and R 4.3.2 (R Foundation for Statistical Computing, Austria; primarily for nomogram development and validation). A two-sided *P* < 0.05 was considered statistically significant.

## Result

### Baseline patient characteristics

**Figure 2:**
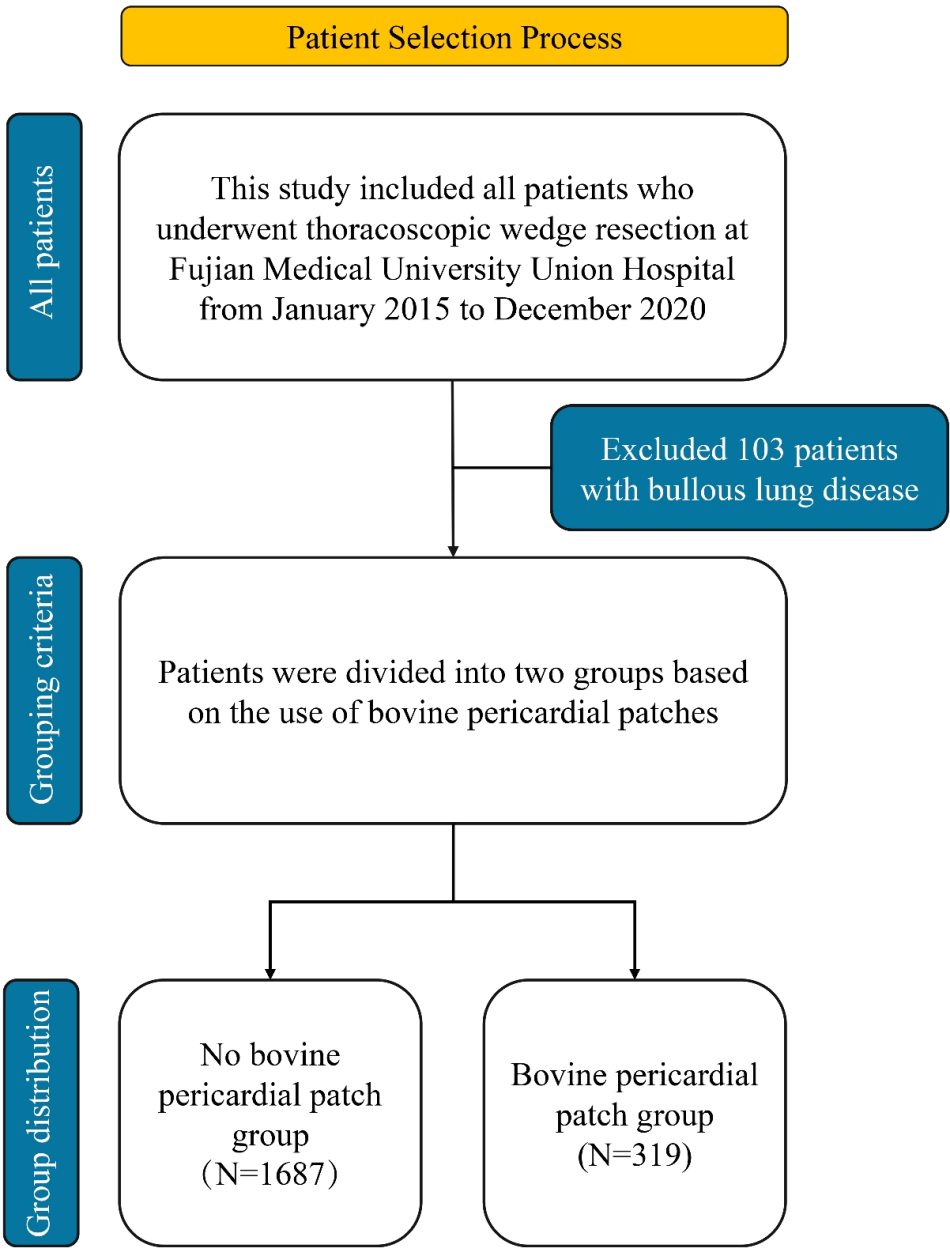
Study population flowchart.

**Figure 3:**
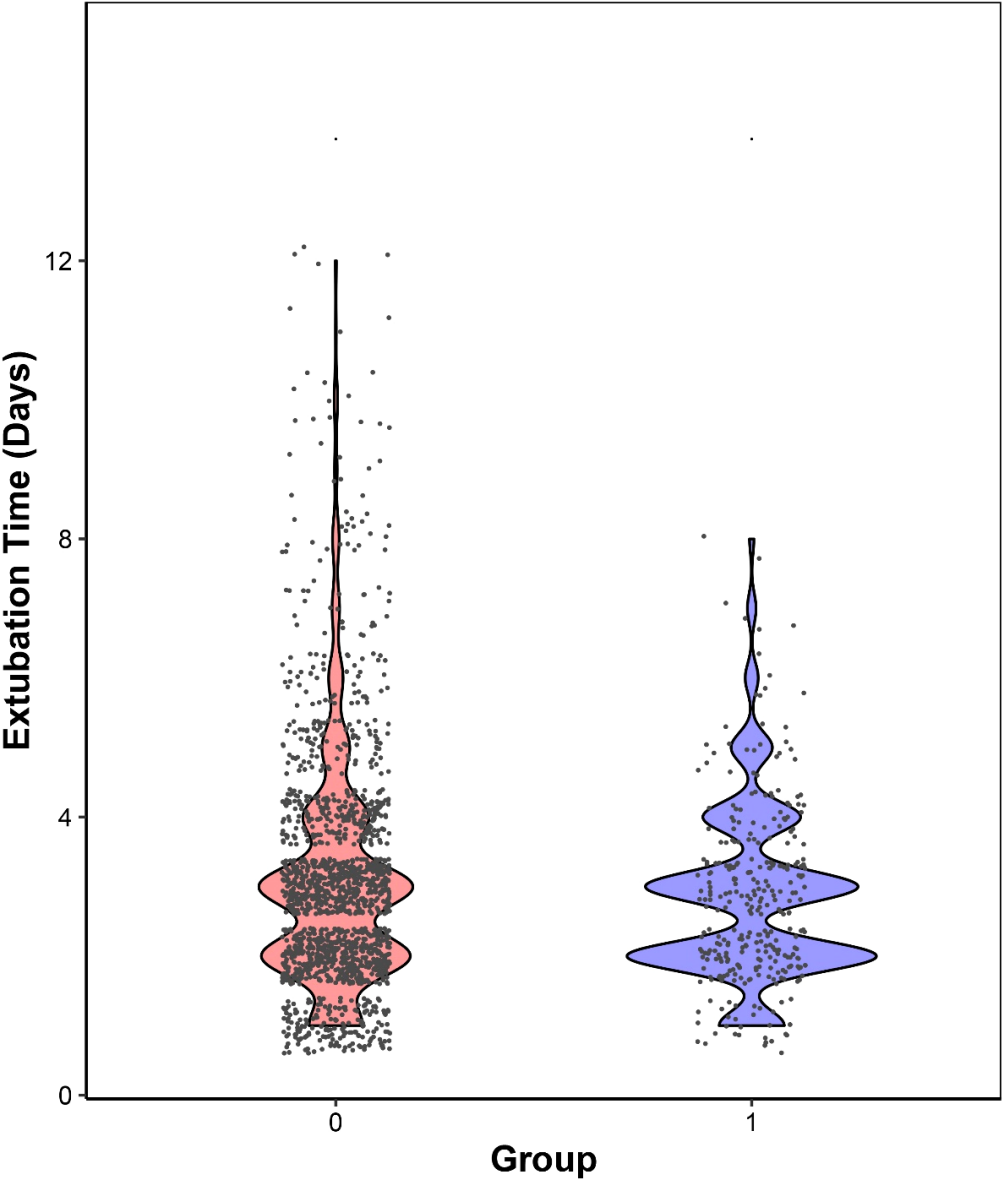
Box plot demonstrating shorter mean extubation time in patch recipients.

A total of 2,006 patients undergoing thoracoscopic wedge resection were included, stratified into two groups: the non-patch group (n=1,687) and the bovine pericardial patch group (n=319) (**Figure 2**). Baseline characteristics were well-balanced between groups, with no significant differences (*P* > 0.05) in age, sex, BMI, smoking/drinking history, hypertension, DM, cardiovascular/respiratory disease history, prior malignancy, thoracic surgery, laterality, multiplicity, ASA classification, emphysema index, operative time, blood loss, thoracic adhesions, and hospital length of stay (**Table 1**). We noted the incidence of PAL was significantly lower in the bovine pericardial patch group (2.8% vs 13.2%; *P* < 0.001). Moreover, the mean extubation time was shorter in the bovine pericardial patch group (2.85 ± 1.27 days vs 3.06 ± 1.67 days; *P* = 0.033), though median was 3 days for both (**Figure 3**).

**Table 1:** Baseline data of the bovine pericardial patch group and the non-bovine pericardial patch group.

Characteristics	Total	Non-bovine pericardial patch group	Bovine pericardial patch group	P-value
	N=2006	N=1687	N=319	
Gender				
Male	1016(50.6%)	851 (50.4%)	165 (51.7%)	0.72
Female	990(49.4%)	836 (49.6%)	154 (48.3%)	
Age				
< 65.00	1587(79.11%)	1335 (79.1%)	252 (79.0%)	1
≥ 65.00	419(20.89%)	352 (20.9%)	67 (21.0%)	
BMI				
< 25.00	1521(75.82%)	1277 (75.7%)	244 (76.5%)	0.817
≥ 25.00	485(24.18%)	410 (24.3%)	75 (23.5%)	
Smoking (%)				
Absent	1232(61.4%)	1036 (61.4%)	196 (61.4%)	1
Present	774(38.6%)	651 (38.6%)	123 (38.6%)	
Drinking (%)				
Absent	1460(72.8%)	1225 (72.6%)	235 (73.7%)	0.75
Present	546(27.2%)	462 (27.4%)	84 (26.3%)	
Hypertension (%)				
Absent	1553(7.4%)	1312 (77.8%)	241 (75.5%)	0.425
Present	453(22.6%)	375 (22.2%)	78 (24.5%)	
Diabetes (%)				
Absent	1720(85.7%)	1446 (85.7%)	274 (85.9%)	1
Present	286(14.3%)	241 (14.3%)	45 (14.1%)	
History of cardiovascular system diseases (%)				
Absent	1918(95.6%)	1613 (95.6%)	305 (95.6%)	1
Present	88(4.4%)	74 ( 4.4%)	14 ( 4.4%)	
History of respiratory system diseases (%)				
Absent	1902(94.8%)	1599 (94.8%)	303 (95.0%)	0.992
Present	104(5.2%)	88 ( 5.2%)	16 ( 5.0%)	
History of malignant tumors (%)				
Absent	1770(88.24%)	1480 (87.7%)	290 (90.9%)	0.128
Present	236(11.76%)	207 (12.3%)	29 ( 9.1%)	
History of thoracic surgery (%)				
Absent	1985(98.9%)	1670 (99.0%)	315 (98.7%)	0.923
Present	21(1.1%)	17 ( 1.0%)	4 ( 1.3%)	
Location (%)				
Left	1199(59.8%)	1004 (59.5%)	195 (61.1%)	0.633
Right	807(40.2%)	683 (40.5%)	124 (38.9%)	
Multiple (%)				
Absent	1638(81.7%)	1376 (81.6%)	262 (82.1%)	0.872
Present	368(18.3%)	311 (18.4%)	57 (17.9%)	
ASA (%)				
0	1116(55.6%)	933 (55.3%)	183 (57.4%)	0.523
1	638(31.8%)	545 (32.3%)	93 (29.2%)	
2	252(12.6%)	209 (12.4%)	43 (13.5%)	
Surgical duration/minutes				
Median (IQR)	80.00(60.00-102.00)	80.00(61.00-102.00)	77.00(60.00-99.00)	0.058
Mean± SD	85.53±35.44	86.00±35.59	81.91±34.53	
Intraoperative blood loss/ml				
Median (IQR)	20.00(15.00-50.00)	20.00(15.00-50.00)	20.00(15.00-40.00)	0.198
Mean± SD	32.52±36.43	32.97±37.62	30.11±29.25	
Thoracic adhesions status (%)				
Absent	1782(88.8%)	1498 (88.8%)	284 (89.0%)	0.981
Present	224(11.2%)	189 (11.2%)	35 (11.0%)	
Air leakage (%)				
Absent	1774(88.4%)	1464 (86.8%)	310±97.2	<0.001
Present	232(11.6%)	223 (13.2%)	9±2.8	
Emphysema index				
Median (IQR)	22.08(20.75-23.36)	22.09(20.74-23.37)	22.04(20.78-23.35)	0.692
Mean ± SD	22.08±1.99	22.09±1.99	22.05±1.93	
Extubation time/days				
Median (IQR)	3.00(2.00-4.00)	3.00(2.00-4.00)	3.00(2.00-4.00)	0.033
Mean ± SD	2.95±1.623	3.06±1.67	2.85±1.27	
Hospital length of stay/days				0.534
Median (IQR)	3.00(2.00-4.00)	3.00(2.00-4.00)	3.00(2.00-4.00)	
Mean ± SD	3.16±2.11	3.17±2.19	3.09±1.59	

### Risk factors for PAL

Univariate logistic regression identified several significant predictors of PAL, which were further refined through multivariate analysis (**Table 2**). Patient demographics and comorbidities played important roles, with age ≥65 years (OR = 2.128, *P* < 0.001) and female gender (OR = 0.654, *P* = 0.003) showing particularly strong associations. Lifestyle factors including smoking (OR = 1.478, *P* = 0.005) and alcohol use (OR = 1.382, *P* = 0.030), along with hypertension (OR = 1.496, *P* = 0.010), were also significantly associated with PAL risk. The most substantial risk factors emerged from surgical history and intraoperative findings. Patients with thoracic adhesions demonstrated a nearly 7-fold increased risk (OR = 6.801, *P* < 0.001), while those with prior thoracic surgery faced an 8.8-fold higher risk (OR = 8.780, *P* < 0.001). Respiratory system disease history increased PAL risk approximately 5-fold (OR = 4.834, *P* < 0.001). Radiological measures also contributed, with each unit increase in emphysema index associated with an 8.4% risk elevation (OR = 1.084, *P* = 0.022). Notably, bovine pericardial patch application showed a strong protective effect, reducing PAL risk by 80.9% (OR = 0.191, *P* < 0.001). These findings were subsequently incorporated into our multivariate model to identify independent predictors.

**Table 2:** Univariate and multivariate logistic regression analysis of air leakage after pulmonary wedge resection.

Variables	Number	Univariate analysis	P value	Multivariate analysis	P value
		OR(95%CI)		OR(95%CI)	
Gender					
Male	1016	Reference	0.003	Reference	0.064
Female	990	0.654(0.494-0.863)		0.720(0.508-1.020)	
Age					
<65	1587	Reference	<0.001	Reference	0.005
≥65	419	2.128(1.576-2.856)		1.619(1.155-2.252)	
BMI					
< 25.00	1521	Reference	0.232		
≥ 25.00	485	0.973(0.93-1.017)			
Smoking (%)					
Absent	1232	Reference	0.005	Reference	0.27
Present	774	1.478(1.121-1.945)		1.207(0.863-1.685)	
Drinking (%)					
Absent	1460	Reference	0.03	Reference	0.669
Present	546	1.382(1.027-1.847)		0.921(0.631-1.339)	
Hypertension (%)					
Absent	1553	Reference	0.01	Reference	0.105
Present	453	1.496(1.098-2.02)		1.326(0.938-1.856)	
Diabetes (%)					
Absent	1720	Reference	0.311		
Present	286	0.807(0.522-1.204)			
History of cardiovascular system diseases (%)					
Absent	1918	Reference	0.103		
Present	88	1.611(0.876-2.779)			
History of respiratory system diseases (%)					
Absent	1902	Reference	<0.001	Reference	0.573
Present	104	4.834(3.127-7.377)		0.842(0.463-1.530)	
History of malignant tumors (%)					
Absent	1770	Reference	0.619		
Present	236	0.895(0.564-1.363)			
History of thoracic surgery (%)					
Absent	1985	Reference	<0.001	Reference	0.228
Present	21	8.78(3.662-21.301)		1.849(0.683-5.146)	
Thoracic adhesions status(%)					
Absent	1782	Reference		Reference	<0.001
Present	224	6.801(4.941-9.344)	<0.001	6.842(4.329-10.736)	
Location (%)					
Left	1199	Reference	0.275		
Right	807	1.166(0.883-1.536)			
Multiple (%)					
Absent	1638	Reference	0.919		
Present	368	0.982(0.681-1.387)			
Bovine pericardial patch					
Absent	1687	Reference		Reference	
Present	319	0.191(0.09-0.354)	<0.001	0.170(0.079-0.322)	<0.001
Emphysema index	2006	1.084(1.012-1.162)	0.022	1.072(0.996-1.154)	0.062

Multivariate analysis incorporating significant univariate predictors identified three key independent factors influencing PAL risk. Thoracic adhesions emerged as the strongest predictor, conferring a nearly 7-fold increased risk (OR = 6.842, 95% CI: 4.329-10.736; *P* < 0.001). Advanced age (≥65 years) moderately elevated risk (OR = 1.619, 95% CI: 1.155-2.252; *P* = 0.005), while bovine pericardial patch use demonstrated a robust protective effect, reducing air leak risk by 83% (OR = 0.170, 95% CI:0.079-0.322; *P* < 0.001). Borderline associations were observed for female gender (OR = 0.720, *P* = 0.064) and emphysema index (OR = 1.072 per unit increase, *P* = 0.062), though these did not reach statistical significance in the final model. Notably, other variables significant in univariate analysis were not retained as independent predictors after multivariate adjustment.

### Nomogram and model validation

**Figure 4:**
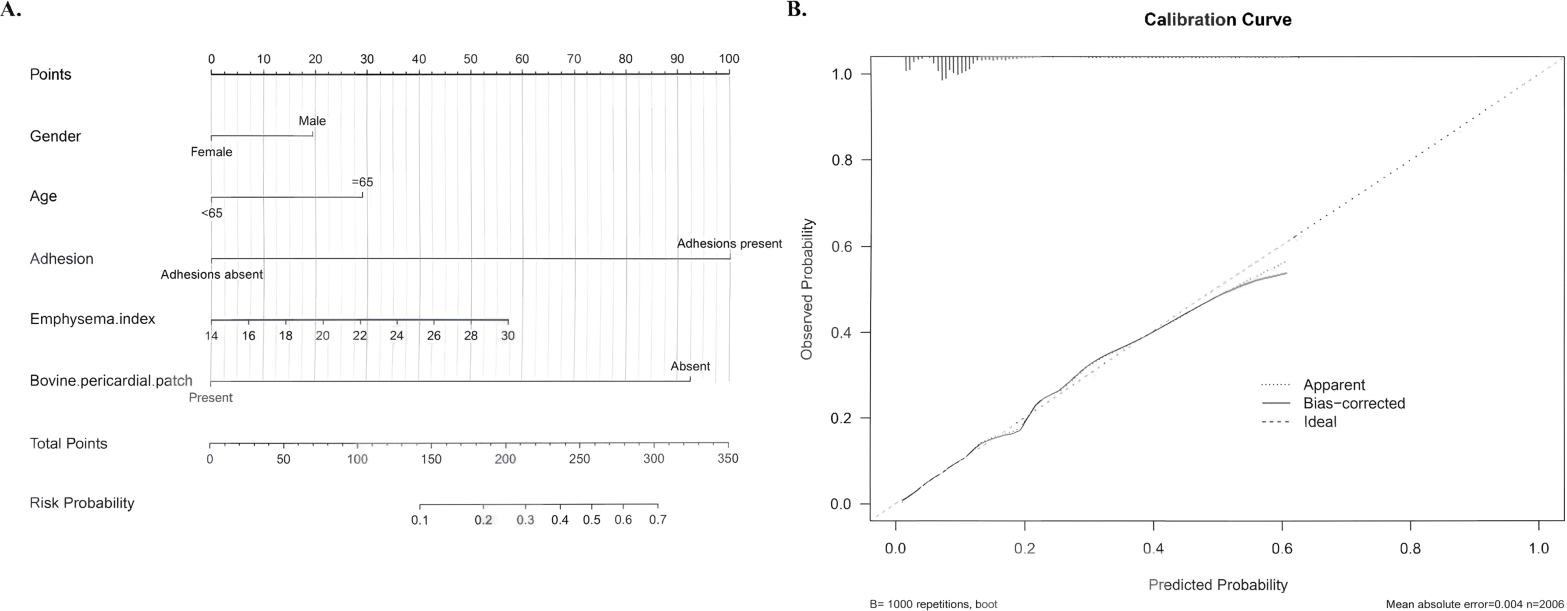
Predictive nomogram development and validation. **(A)** Nomogram assigning weighted points to independent predictors: thoracic adhesions, patch use, emphysema index, and age ≥65 years. **(B)** Calibration plot showing excellent agreement between predicted and observed PAL probabilities, with bootstrap-corrected estimates closely tracking the ideal line.

The predictive nomogram incorporated all variables with P<0.1 in multivariate analysis (**Figure 4A**). Thoracic adhesions demonstrated the strongest contribution (100 points), followed closely by bovine pericardial patch use (92.5 points, protective effect). Emphysema index (57.5 points) and age ≥65 years (30 points) showed moderate predictive weights. Calibration curves revealed excellent agreement between predicted and observed probabilities, with both apparent and bias-corrected (1000 bootstrap samples) models closely following the 45° ideal line (**Figure 4B**), indicating robust model reliability.

### Model performance and clinical utility

**Figure 5:**
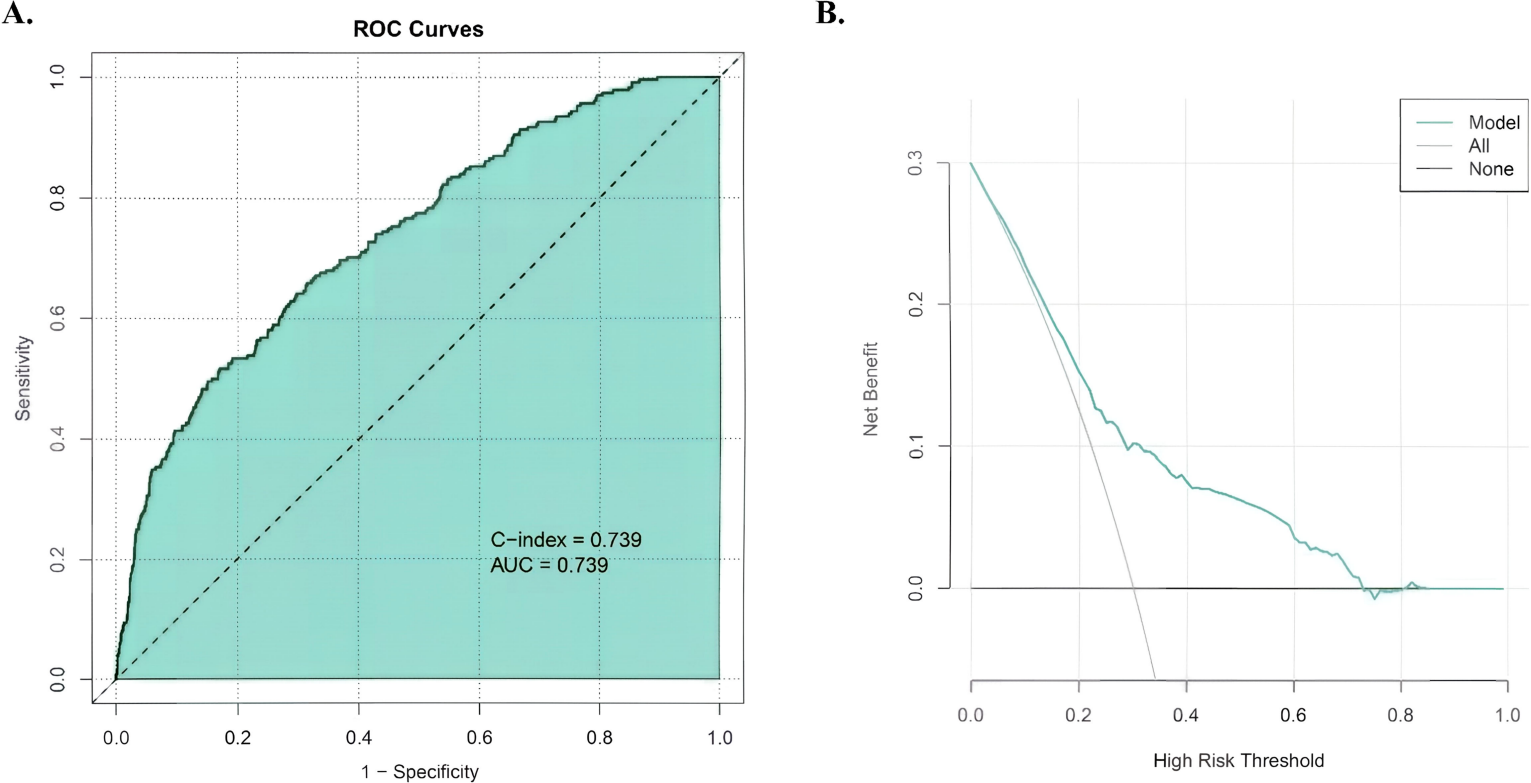
Model performance metrics. **(A)** ROC curve demonstrating moderate-to-good discrimination (AUC = 0.739). **(B)** Decision curve analysis showing superior net benefit of the patch-based strategy versus “treat-all” or “treat-none” approaches across 20-80% risk thresholds, with peak net benefit (0.2) at 40% clinical decision threshold.

The prediction model demonstrated moderate-to-good discriminative ability, with an AUC of 0.739 in ROC analysis (**Figure 5A**) that matched the C-index. Decision curve analysis (**Figure 5B**) confirmed the clinical value of the bovine pericardial patch strategy, showing superior net benefit across threshold probabilities of 20-80%. At a typical clinical threshold of 40% risk, the net benefit reached 0.2, suggesting this approach would optimize decision-making for most patients undergoing wedge resection.

## Discussion

To our knowledge, this represents the first comprehensive evaluation of bovine pericardial patch application in thoracoscopic wedge resection for PAL prevention. Our findings demonstrate that patch utilization significantly reduces PAL incidence and decreases chest tube duration. Combined with the identification of key risk factors and development of a validated predictive model, these results provide a valuable framework for optimizing perioperative management in patients undergoing limited pulmonary resection.

This study demonstrates that bovine pericardial patches provide significant protection against PAL, reducing risk by 83% in multivariate analysis. Their efficacy appears comparable or superior to existing options like fibrin sealants and PGA patches (14, 15). Building on established evidence of their excellent biological properties in vascular reconstruction (sealing capacity and absorbability) (19, 20), our findings position bovine pericardial patches as a cost-effective alternative. The protective mechanism likely involves reinforcement of staple lines-particularly in fragile lung parenchyma-preventing gas leakage from compromised alveoli or small airways (21). The observed reduction in chest tube duration (2.85 vs 3.06 days) further supports their role in enhanced recovery, aligning well with ERAS (Enhanced Recovery After Surgery) principles.

This study identified three independent predictors of PAL: thoracic adhesions, advanced age (≥65 years), and bovine pericardial patch use, providing crucial evidence for preoperative risk stratification. Thoracic adhesions emerged as the strongest risk factor (OR 6.84). This may be attributable to two mechanisms: first, anatomical distortion increases the risk of parenchymal injury during resection; second, impaired pulmonary compliance may compromise staple line integrity (22). Advanced age conferred a 61.9% increased risk, potentially attributable to reduced lung tissue elasticity in elderly patients and diminished wound healing capacity (23, 24).

It is noteworthy that variables significant in univariate analysis (e.g., smoking, hypertension, history of respiratory disease) were not included in the multivariate model, suggesting their effects may be mediated through other variables. For instance, smoking might increase the risk of air leakage by inducing emphysema and weakening lung parenchymal strength (25). However, after adjusting for stronger confounders such as pleural adhesions, its independent effect was masked. This finding underscores the necessity of multivariate analysis in distinguishing independent risk factors from confounding factors.

The nomogram constructed in this study integrates key predictive factors, including pleural adhesion, age, emphysema index, and patch use, with a C-index of 0.739, demonstrating good discriminative ability. Internal validation indicated high model stability, and calibration curve analysis revealed excellent agreement between predicted and observed risks. Decision curve analysis further confirmed that the model provided significant clinical net benefit within the 20%-80% risk threshold range, outperforming the traditional “treat-all” or “treat-none” strategies. This tool offers clinicians a quantitative method for risk assessment, enabling personalized treatment decisions. For high-risk patients, targeted use of bovine pericardial patches can effectively reduce postoperative air leakage. For low-risk patients, unnecessary interventions can be avoided, thereby lowering healthcare costs and minimizing potential complications. The application of this model facilitates optimized allocation of medical resources, reduces overtreatment, and ensures precise interventions for high-risk patients, aligning with the principles of precision medicine and individualized therapy.

This study has several limitations that warrant careful consideration. First, the retrospective study design, conducted as a single-center analysis, inherently limits causal inferences and generalizability. The decision to use the patch was selective and surgeon-dependent, potentially influenced by surgeon experience, intraoperative findings, and some clinical factors that were not fully captured in the records, which may introduce selection bias and potential surgeon-related bias. This contributes to limited external generalizability, as our findings reflect practices at one institution and may not apply to diverse patient populations or settings. Although we performed appropriate statistical adjustments, residual confounding may still exist. Second, the unequal sample sizes between groups, while reflecting real-world practice, may affect the stability of effect estimates and limit the power of some subgroup analyses. Third, this study primarily evaluated short-term postoperative outcomes, and longer-term complications and prognosis were not assessed. In addition, we did not further stratify outcomes by final postoperative pathology; future studies with larger cohorts could explore whether the observed benefit is consistent across pathological subtypes. Furthermore, while the predictive model underwent internal validation using bootstrapping (yielding an AUC of 0.739; 95% CI: 0.702-0.776), calibration plots, and decision curve analysis (demonstrating superior net benefit across 20-80% threshold probabilities), the lack of external validation in independent multicenter datasets restricts its broader applicability. Therefore, prospective, multicenter studies are warranted to further validate these findings and improve generalizability.

## Conclusion

As an adjunct to thoracoscopic wedge resection, bovine pericardial patches significantly reduce the risk of PAL and improve recovery outcomes. The risk factors and predictive model identified in this study facilitate targeted intervention and optimized postoperative management. Given its balanced benefits in efficacy, safety, and cost-effectiveness, bovine pericardial patches should be prioritized for high-risk PAL patients, particularly those with pleural adhesions or advanced age. Future multicenter prospective randomized controlled trials are warranted to validate their clinical efficacy and evaluate long-term cost-effectiveness. Further research should explore their sealing mechanisms and potential applications in complex procedures.

## Data Availability

The original contributions presented in the study are included in the article/supplementary material. Further inquiries can be directed to the corresponding authors.
